# Quality of life impact of mental health conditions in England: results from the adult psychiatric morbidity surveys

**DOI:** 10.1186/1477-7525-12-6

**Published:** 2014-01-14

**Authors:** Jennifer Roberts, Pamela Lenton, Anju D Keetharuth, John Brazier

**Affiliations:** 1 Department of Economics, University of Sheffield, Sheffield, UK; 2 School of Health and Related Research, University of Sheffield, Sheffield, UK

**Keywords:** EQ-5D, SF-6D, Health-related quality of life, Health state utility values, Mental health, Co-morbidities, Depression, Anxiety

## Abstract

**Background:**

The main objective is to present health state utility estimates for a broad range of mental health conditions including anxiety, depression, long-term depression, obsessive compulsive disorder, phobia, panic disorder, psychosis, alcohol and drug dependency that can be used in economic models.

**Methods:**

This study uses pooled data from the Adult Psychiatric Morbidity Surveys carried out in 2000 and 2007 of a representative sample of the general population in England. Health state utility values measured by the SF-6D and EQ-5D indices are the dependent variables. Independent variables include background characteristics, mental health and physical health conditions. Regression models were estimated using OLS for the SF-6D and tobit for EQ-5D. Further regressions were carried out to consider the impact of mental health and physical health morbidities and the impact of severity of conditions on utility values.

**Results:**

Mental health conditions tend to have a larger impact on health state utility values than physical health conditions. The mental health conditions associated with the highest decrements in utility are: depression, mixed anxiety and depressive disorders and long-term depression. Interaction terms used to model the effect of co-morbidities are generally found to be positive implying that simply adding the utility decrements for two mental health conditions overestimates the burden of the disease.

**Conclusions:**

This paper presents reliable and representative community based mean SF-6D and EQ-5D estimates with standard errors for health state utility values across a broad range of mental health conditions that can be used in cost effectiveness modelling.

## Background

While the prevalence of mental health conditions is increasing in the UK, there is a dearth of country specific data on the impact of mental health conditions on health-related quality of life (HRQoL). There are a number of European studies that investigate HRQoL decrements as a result of mental health conditions but none of these includes the UK [1-3]. The focus of many surveys has been epidemiological in nature concentrating on prevalence of mental health conditions. Health state utility values (HSUVs) take the form of an index constructed from an outcome measure normally anchored from zero to one to represent death and full health respectively. They are normally used by decision-makers to evaluate and compare cost-effectiveness of various treatments. HSUVs are particularly scarce for more complex conditions like psychosis, phobia and panic disorder [4] and disutility associated with different levels of severity.

Decision makers such as the National Institute for Health and Care Excellence (NICE) in the UK recommend that the results of economic evaluations in healthcare are presented in terms of quality adjusted life years (QALYs) which are a composite measure of health related quality of life and life expectancy. The most commonly used measures for putting the ‘Q’ into the QALY are generic preference-based measures of health, such as the EQ-5D [5] and SF-6D [6].This paper seeks to address the lack of available HSUVs associated with mental health conditions by providing estimates that can be used in economic evaluation from a representative community based sample.

Another important feature of mental health conditions is the common existence of co-morbidities, arising from other mental health conditions as well as physical ones. Understanding the impact of one condition on HSUVs cannot be done in isolation, but only after controlling for other health conditions. Previous studies of mental health conditions have largely failed to do this. There are several studies in the literature that highlight the lack of consensus on how best to calculate HSUVs [7,8]. While attempts have been made to calculate HSUVs for co-morbid physical conditions [9], for example by adding or multiplying the effects of separate conditions, there is still overall very little empirical research on co-morbidities for those with mental health conditions.

### Aims of the study

This paper has three objectives: (i) to estimate the impact of mental health disorders on quality of life in England (ii) to compare and contrast the impact of physical health and mental health co-morbidities on quality of life (iii) to assess the impact of severity of depression, anxiety, phobia and panic disorders on quality of life.

Specifically we estimate the decrement associated with various mental health disorders using the SF-6D and EQ-5D indices from a representative sample of the UK general population and controlling for a wide range of background variables.

## Methods

### Data

We used health-related quality of life data and information on health status collected in the Adult Psychiatric Morbidity Survey (APMS) carried out in 2000 and 2007 [10,11]. This is a rigorously conducted general population survey aiming to provide information on the prevalence of psychiatric conditions among people living in Great Britain, as well as their associated social disabilities and use of services. Each APMS recruited about 8,000 working age adults in private households. APMS is unique in the UK for having data on a broad range of conditions including common mental health disorders like depression, anxiety and obsessive compulsive disorder, psychotic conditions, personality disorders and alcohol and drug dependence. They also contain general health measures including the SF-12 health index, a measure from which the SF-6D preference based utility index can be obtained [12]. There is also information on socio-demographic data, education and employment, income and debt, accommodation and stressful life events. While the 2000 survey covered England, Wales and Scotland, APMS 2007 only interviewed people in England so our analysis uses data for England only.

In both years APMS interviews are conducted in two stages. First, a computer assisted personal interview in the respondents own home covering neurotic symptoms and disorders using the Clinical Interview Schedule Revised (CIS-R) and screening items on personality disorder and psychosis. CIS-R is a structured interview that has been standardised so that it can be administered by social survey interviewers. It enquires about 14 common neurotic symptoms allowing categorisation according to ICD-10 criteria. CIS-R also measures the severity of the condition and is widely used [13,14]. A second stage sample was chosen comprising respondents who satisfied screening criteria for psychotic and personality disorder. The second stage interviews were conducted by trained psychologists using Schedules for Clinical Assessment in Neuropsychiatry (SCAN) and Structured Clinical Interview (SCID-II). In 2000 (2007) there were 8580 (7461) initial interviews, a response rate of 54 (57)%. At the second stage there were 638 (630) interviews and a response rate of 73 (74)%. Our analysis sample comprises 5688 individuals in 2000 and 5388 individuals in 2007. Data for 2000 and 2007 are pooled.

### Measuring HSUVs

Two generic preference-based measures were derived from the SF-12 data: SF-6D and the EQ-5D. The SF-6D health utility index is derived from individual responses to the SF-12, a generic health measure based on items taken from the SF-36 health survey, a standardised questionnaire used to assess patient health [15]. Brazier and Roberts [9] developed a preference based index for the SF-12 using an algorithm estimated from standard gamble valuations of a sample of SF-6D states obtained from members of the UK general population [6]. The index takes values that range from zero (equivalent to dead) to one (full health).

The EQ-5D questionnaire consists of 5 simple questions on mobility, self-care, usual activities, pain and discomfort, anxiety and depression. As raw data was not collected from respondents using the EQ-5D questionnaire, EQ-5D scores have been generated through mapping from SF12 items using a response approach mapping [16]. It is deemed important to present the utilities using the EQ-5D, since the former is the method preferred by NICE in the reference case [17]. In the response mapping approach, multinomial logistic regression is used to estimate the probability that a respondent will select a particular level of response to questions in the EQ-5D, using individual question responses from the SF-12 as predictors. EQ-5D responses are predicted using Monte Carlo simulation methods and UK tariffs are then applied to the raw scores to generate the EQ-5D index [5].

### Measuring severity of conditions

It is often useful to know the severity of the condition, as quality of life decrements and health care costs are usually much higher for greater levels of morbidity. Each condition is diagnosed via a set of four questions from the CIS-R. For example in the case of anxiety these are: (i) felt generally anxious/nervous/tense for 4 days or more in the past seven days; (ii) in past seven days anxiety/nervousness/tension has been very unpleasant; (iii) in the past seven days have felt any of the following symptoms when anxious/nervous/tense (Racing heart, sweating or shaking hands, feeling dizzy, difficulty getting one’s breath, dry mouth, butterflies in stomach, nausea or wanting to vomit); (iv) felt anxious/nervous tense for more than three hours in total on any one of the past seven days. Each question scores one if that symptom was present, giving a total anxiety score ranging from zero for no symptoms to four. This score can be used as a measure of severity of the condition. A similar approach was taken with depression, panic and phobia.

### Analysis

The basic model to be estimated is:

(1) $$U_{i}=f\left(M_{i},P_{i},X_{i,}\epsilon_{i}\right)$$

Where *U*_*i*_ is health utility for respondent *i*; *M* is mental health, *P* is physical health and *X* is a set of background characteristics, ϵ_i_ is a random error term.

Two separate sets of analyses are carried out with SF-6D and EQ-5D as dependent variables. The initial modelling was undertaken using Ordinary Least Squares (OLS) but this can be criticised. OLS estimates are biased and inconsistent, because both the SF-6D and EQ-5D distributions are skewed and in addition the EQ-5D distribution is truncated with many observations at the upper value of one [18]. To overcome these problems, OLS models for SF-6D are estimated with robust error variance as Breusch-Pagan/Cook-Weisberg tests results suggest heteroskedastic errors, and for the EQ-5D, tobit models are used to deal with the truncated nature of the data.

Mental health (*M*) is measured by a set of dichotomous variables to represent the presence of specific disorders. Diagnosis of specific disorders were assigned by the Office of National Statistics using answers to various sections of the CIS-R and applying algorithms based on the ICD-10 diagnostic criteria; these disorders are: generalised anxiety disorder (GAD), mixed anxiety depressive disorder (MADD), panic disorder, obsessive compulsive disorder (OCD), phobia, and depression. Psychosis and personality disorder are assigned via the Stage 1 screening questions, alcohol dependence is defined according to the Severity of Alcohol Dependence questionnaire (SAD-Q) and drug dependence defined according to questions used in the US Epidemiologic Catchment Area survey. A variable was generated to represent people who self-report that they have long-term depression lasting for a period of 2 years or over [19]. More detail on definitions for each disorder can be found in the APMS technical reports [10,11].

Physical health (*P*) is measured by a set of dichotomous variables denoting the presence of self-reported long-standing health conditions: muscular-skeletal, respiratory, digestive, heart and circulatory, urinary, skin, ear, eye, neoplasm, blood disorder, and infection. The set of background variables (*X*) include age, marital status, presence of children aged 16 or under in the household, employment status, ethnicity, education and income. Dummy variables for regions and year are also included. A full list of variables and definitions can be obtained from the corresponding author.

Although co-morbidities are an important issue for cost-effectiveness modelling, there is no consensus about the best method to estimate HSUVs for co-morbidities [7]. To allow a flexible approach we have explored all first order interactions between mental health conditions and physical health conditions by estimating models (2) and (3) below:

(2) $$U_{i}=f\left(M_{i},P_{i},X_{i,}IM_{i},\epsilon_{i}\right)$$

(3) $$U_{i}=f\left(M_{i},P_{i},X_{i,}IP_{i,}\epsilon_{i}\right)$$

Where *IM* is a set of dichotomous variables representing first order interactions between the seven mental health conditions described above and *IP* is a set of dichotomous variables representing first order interactions between the seven mental health conditions and the seven physical health conditions described above. It was not possible to investigate co-morbidities for personality disorder, psychosis and panic due to the small number of observations. In addition some physical conditions could not be considered due to small sample sizes, including ear complaints, neoplasm, blood disorder and infectious disorder. Data from the APMS was downloaded from the UK Data Archive [20] and STATA 11 was used for the analysis.

## Results

Table 1 reports descriptive statistics for the entire sample of 11080 cases. The distribution of the SF-6D health utility index is negatively skewed with a concentration at the higher end of the scale (Additional file 1: Figure S1a). However there is no obvious ceiling or floor effect and only a small proportion of observations take the value of one (less than 5%). While the mean scores for SF-6D (0.791) and EQ-5D (0.808) are very similar, the distributions are very different. The EQ-5D distribution has a ceiling effect with 33% of observations at full health. SF-6D and EQ-5D scores range between 0.35 and 1 and between -0.38 and 1 respectively (Additional file 1: Figure S1b).

**Table 1:** Descriptive statistics for entire sample

	**N**	**%**
Total sample	11080	
Male	4893	44.2
Single	2405	21.7
Married	6796	61.3
widow/divorced/separated	1879	17.0
Children under 16	3786	34.2
Education- Degree	2151	19.4
Education- HND/Teach/Nursing	854	7.7
Education - A level	1680	15.2
Education - GCSE/O level	3048	27.5
Education - lower level	760	6.9
Education - none	2448	22.1
Gross personal income < £5200 p.a.	2218	20.0
Gross personal income 5200-10399 p.a.	2118	19.1
Gross personal income 10400 -15559 p.a.	1781	16.1
Gross personal income 15560 m-20799 p.a.	1278	11.5
Gross personal income 20800 -33799 p.a.	1885	17.0
Gross personal income >33800 p.a.	1074	9.7
Non white	1084	9.8
Working	7794	70.3
	**Mean**	**Standard deviation**
Age in years	42.7	12.8
SF-6D index	0.791	0.139
EQ-5D index	0.808	0.206

Table 2 shows the descriptive statistics on physical and mental health conditions with mean utility scores. Around 82% of people in this dataset have no mental health disorder, similar to the prevalence rate reported in the APMS reports [10,11]. The most common problem is Mixed Anxiety Depressive disorder (MADD) found in around 10% of respondents, and this is followed by alcohol dependency, which is here defined as any level of dependency detected by the SADQ, ranging from mild to severe. The SF-6D (EQ-5D) scores for those with no physical health problem and no mental problem are 0.829 (0.851) and 0.827 (0.842) respectively. All the scores for the EQ-5D are marginally higher than the SF-6D. Long-term depression and depression sufferers have the lowest SF-6D (EQ-5D) scores of 0.532 (0.513) and 0.551 (0.537) respectively. According to the neurotic symptoms score, those with more severe symptoms have monotonically lower utility scores as measured by both SF-6D and EQ-5D.

**Table 2:** Descriptive statistics: utility scores for people with physical and mental health conditions

***Physical health***	***n***	***%***	***SF-6D mean***	***SF-6D sd***	***EQ-5D mean***	***EQ-5D sd***
No physical health problems	5879	53.1	0.829	0.114	0.851	0.165
Muscular/skeletal complaint	2471	22.3	0.709	0.160	0.705	0.258
Respiratory complaint	969	8.7	0.726	0.159	0.740	0.254
Digestive complaint	606	5.5	0.706	0.163	0.710	0.261
Heart/circulatory complaint	1232	11.1	0.732	0.162	0.741	0.251
Urinary related complaint	588	5.3	0.689	0.159	0.696	0.276
Skin complaint	726	6.6	0.747	0.147	0.763	0.225
Ear complaint	434	3.9	0.737	0.154	0.752	0.247
Eye complaint	819	7.4	0.756	0.152	0.771	0.235
Neoplasm	117	1.1	0.689	0.160	0.710	0.261
Blood disorder	214	1.9	0.725	0.165	0.722	0.275
Infectious disorder	52	0.5	0.681	0.142	0.702	0.240
*Mental health*						
No mental health problems*	8263	74.6	0.827	0.114	0.842	0.170
Generalised anxiety disorder	613	5.5	0.626	0.141	0.643	0.288
Mixed anxiety depressive disorder	1098	9.9	0.657	0.131	0.681	0.258
Panic disorder	121	1.1	0.645	0.134	0.664	0.274
Obsessive compulsive disorder	162	1.5	0.576	0.113	0.593	0.294
Phobia	267	2.4	0.573	0.114	0.577	0.297
Depression	367	3.3	0.551	0.105	0.537	0.311
Long-term depression	187	1.7	0.532	0.104	0.513	0.324
Psychosis	81	0.7	0.623	0.138	0.665	0.281
Personality disorder	31	0.3	0.648	0.153	0.706	0.274
Alcohol dependency (any)	781	7.0	0.761	0.144	0.790	0.222
Drug dependency	379	3.4	0.732	0.144	0.774	0.216
*Neurotic symptoms score*						
cisr score_1 (0 - 5)	7146	64.5	0.848	0.100	0.861	0.152
cisr score_2 (6 - 11)	1954	17.6	0.744	0.126	0.774	0.205
cisr score_3 (12 -17)	957	8.6	0.679	0.125	0.712	0.237
cisr score_4 (18 -23)	489	4.4	0.617	0.125	0.636	0.273
cisr score_5 (24 –29)	282	2.5	0.572	0.112	0.576	0.291
cisr score_6 (30 –35)	161	1.5	0.542	0.098	0.536	0.306
cisr score_7 (36+)	91	0.8	0.518	0.099	0.464	0.338

*No mental health problem category includes a score of 11 or below on the cis-r scale.

The results from the OLS and tobit regressions for SF-6D and EQ-5D respectively are shown in Table 3. Marginal effects have been calculated at the means of the explanatory variables for tobit regressions. The highest decrements in HSUVs are observed for depression, MADD, long-term depression and GAD for both SF-6D and EQ-5D. The HSUV decrements in SF-6D (EQ-5D) scores are as follow: depression -0.137 (-0.159); MADD -0.136 (-0.127); long-term depression -0.100 (-0.118); GAD -0.086 (-0.070); phobia -0.083 (-0.081); panic -0.077 (-0.076); OCD -0.057 (-0.034) and; drug dependency -0.027 (-0.011). The three mental health conditions that do not have a statistically significant effect on health utility are psychosis, personality disorder and alcohol dependence. The coefficients for psychosis and personality disorder may be insignificant due to the small numbers of people (81 and 31 respectively) suffering from these conditions. All of the physical health conditions, except eye complaints, are statistically significant at 1% level of significance. In the models for both SF-6D and EQ-5D, socio-demographic characteristics were controlled for. They are not reported in this paper but are available by request from authors.

**Table 3:** Health decrements in SF-6D and EQ-5D scores

	**SF-6D (OLS regression)**			**EQ-5D (Tobit regression)**		
	**Coefficient**		**SE**	**Coefficient**		**SE**
male	0.011	***	0.002	0.015	***	0.004
age	-0.005	***	0.001	-0.002	**	0.001
**Mental health conditions**						
Generalised anxiety disorder	-0.086	***	0.006	-0.070	***	0.009
Mixed anxiety depressive disorder	-0.136	***	0.004	-0.127	***	0.007
Panic disorder	-0.077	***	0.012	-0.076	***	0.019
Obsessive compulsive disorder	-0.057	***	0.011	-0.034	**	0.016
Phobia	-0.083	***	0.008	-0.081	***	0.015
Depression	-0.137	***	0.007	-0.159	***	0.014
Long-term depression	-0.100	***	0.01	-0.118	***	0.019
Psychosis	-0.001		0.011	0.017		0.02
Personality disorder	-0.010		0.024	0.013		0.032
Alcohol dependency (any)	-0.002		0.004	0.006		0.007
Drug dependency	-0.027	***	0.006	-0.011		0.01
**Physical health conditions**						
Muscular/skeletal complaint	-0.071	***	0.003	-0.093	***	0.005
Respiratory complaint	-0.029	***	0.004	-0.025	***	0.007
Digestive complaint	-0.030	***	0.005	-0.032	***	0.008
Heart/circulatory complaint	-0.028	***	0.004	-0.025	***	0.006
Urinary related complaint	-0.040	***	0.005	-0.037	***	0.009
Skin complaint	-0.016	***	0.004	-0.019	**	0.008
Ear complaint	-0.028	***	0.006	-0.016		0.01
Eye complaint	-0.006		0.004	-0.004		0.007
Neoplasm	-0.054	***	0.012	-0.046	**	0.019
Blood disorder	-0.034	***	0.009	-0.039	***	0.015
Infectious disorder	-0.050	***	0.018	-0.037		0.028
Constant	0.881	***	0.013			
Observations	10,289					
R-squared/Pseudo R-squared	0.413			0.252		
Reset test (omitted variables)	79.07	***				
Pregibon link test (model specification)	0.348	***	0.084	0.108		
Chi-square				2153	***	

Note: Socio-demographics variables including education, income level, ethnicity, working status and regions have been controlled for in the model but are not reported here. ***p < 0.01, **p < 0.05, *p < 0.1.

For the OLS regression for SF-6D, the adjusted R^2^ is 0.41 but the Ramsey Regression Equation Specification Error Test (RESET) [21] and Pregibon [22] link test statistics suggest that the model suffers from omitted variables and misspecification problems respectively. It is worth noting that these tests also revealed misspecification problems with both tobit [23] and Postestimation Generalized Linear Models (PGLM) models [24,25] which were also explored for the SF-6D (not reported here). The marginal effects of physical and mental health conditions on the SF-6D index are very similar regardless of the estimation procedure and functional form chosen, a finding similar to that of Jones [26] who compared a number of different models for modelling health care cost data. In the case of EQ-5D as the dependent variable, the link test suggests that the model is correctly specified making tobit regression a better model than OLS in this case.

### Co-morbid Health Conditions

Table 4 reports the results for the models exploring interactions between mental health conditions. Only the coefficients on the main mental health conditions and the significant (at p = 0.05) interactions are reported. The coefficients on the mental health conditions are different compared to Table 3 but remain statistically significant. The first point to note is that the coefficients on all of the interaction terms are positive implying that having both conditions results in a health utility decrement that is less than the sum of the individual coefficients. For example, having GAD and depression, the decrement to the SF-6D index is -0.107 - 0.182 + 0.122 = -0.167; this is smaller than that suggested by the additive model of -0.289. In this case it is also highlighted that the interaction term more than offsets the main effects of GAD. Similarly using EQ-5D, the decrement associated with GAD and depression is -0.213. Despite the insignificant main effects for alcohol dependence, there are significant positive interactions with GAD, MADD (EQ-5D only) and depression.

**Table 4:** Utility values for mental health co-morbidities: SF6 and EQ-5D scores

		**SF 6D OLS models**			**EQ-5D tobit models**		
	**N**	**Coefficient**		**se**	**Coefficient**		**se**
Generalised anxiety disorder		-0.094	***	0.006	-0.081	***	0.010
OCD		-0.099	***	0.012	-0.091	***	0.023
GAD X OCD	70	0.103	***	0.019	0.097	***	0.019
Generalised anxiety disorder		-0.101	***	0.006	-0.081	***	0.010
Phobia		-0.133	***	0.010	-0.115	***	0.019
GAD X Phobia	124	0.121	***	0.015	0.063	***	0.019
Generalised anxiety disorder		-0.107	***	0.006	-0.086	***	0.011
Depression		-0.182	***	0.007	-0.195	***	0.018
GAD X Depression	162	0.122	***	0.013	0.068	***	0.016
Generalised anxiety disorder		-0.094	***	0.006	-0.078	***	0.000
Alcohol Dependency		-0.008	*	0.051	-0.0001		0.008
GAD X Alcohol Dependency	90	0.057	***	0.015	0.050	***	0.018
Generalised anxiety disorder		-0.090	***	0.006	-0.076	***	0.010
Drug Dependency		-0.033	***	0.006	-0.019	*	0.011
GAD X Drug Dependency	48	0.057	***	0.021	0.064	***	0.023
MADD		-0.138	***	0.004	-0.132	***	0.008
Alcohol Dependency		-0.006		0.004	-0.001		0.008
MADD X Alcohol dependency	111	0.024	*	0.012	0.044	***	0.017
OCD		-0.096	***	0.012	-0.072	***	0.021
Phobia		-0.105	***	0.009	-0.102	***	0.016
OCD X Phobia	56	0.133	***	0.020	0.091	***	0.021
OCD		-0.110	***	0.013	-0.096	***	0.024
Depression		-0.155	***	0.007	-0.181	***	0.015
OCD X Depression	78	0.127	***	0.019	0.101	***	0.018
Phobia		-0.127	***	0.010	-0.090	***	0.018
Depression		-0.165	***	0.008	-0.165	***	0.016
Phobia X Depression	111	0.126	***	0.016	0.023		0.023
Depression		-0.144	***	0.007	-0.151	***	0.015
Alcohol Dependency		-0.005		0.004	0.009		0.007
Depression X Alcohol Dependency	73	0.040	**	0.017	-0.040		0.028

***p < 0.01, **p < 0.05, *p < 0.1.

Table 5 reports the results to explore interactions with physical conditions; only for models where the coefficients on the main health conditions and the interactions are significant (at p = 0.05). The coefficients on the mental and physical health conditions are similar to those in Table 3 and are statistically significant. Of the interactions reported, the majority are positive, meaning that the presence of both disorders reduces health utility by a smaller amount than that suggested by the additive model. The only interaction terms that are negative are MADD and respiratory complaints, and MADD and muscular skeletal complications with the latter interaction being insignificant.

**Table 5:** Utility values for mental health and physical health co-morbidities: SF6 and EQ-5D scores

		**SF 6 OLS models**			**EQ-5D tobit models**		
	**N**	**Coefficient**		**se**	**Coefficient**		**se**
Generalised anxiety disorder		-0.095	***	0.006	-0.077	***	0.012
Muscular/skeletal complaint		-0.073	***	0.003	-0.094	***	0.005
GAD X muscular/skeletal	233	0.025	**	0.011	0.015		0.015
Generalised anxiety disorder		-0.090	***	0.006	-0.075	***	0.010
Digestive complaint		-0.034	***	0.006	-0.036	***	0.009
GAD X digestive complaint	74	0.034	*	0.017	0.03		0.021
Generalised anxiety disorder		-0.091	***	0.006	-0.071	***	0.010
Urinary complaint		-0.044	***	0.006	-0.038	***	0.009
GAD X Urinary complaint	75	0.039	***	0.018	0.008		0.023
Generalised anxiety disorder		-0.091		0.006	-0.079	***	0.010
Eye complaint		-0.009	**	0.004	-0.009		0.007
GAD X eye complaint	71	0.039	**	0.018	0.06	***	0.019
MADD		-0.133	***	0.004	-0.125	***	0.008
Respiratory complaint		-0.025	***	0.004	-0.023	***	0.007
MADD X respiratory complaint	150	-0.025	**	0.010	-0.013		0.017
MADD		-0.139	***	0.004	-0.126	***	0.007
Urinary complaint		-0.045	***	0.006	-0.035	***	0.010
MADD X Urinary complaint	113	0.028	**	0.013	-0.009		0.020
MADD		-0.139	***	0.004	-0.132	***	0.007
Skin complaint		-0.020	***	0.005	-0.027	***	0.008
MADD X skin complaint	97	0.031	**	0.013	0.049	***	0.018
MADD		-0.136	***	0.004	-0.121	***	0.009
Muscular/skeletal complaint		-0.071	***	0.003	-0.090	***	0.005
MADD X muscular/skeletal	369	-0.001		0.008	-0.016		0.013
Depression		-0.154	***	0.008	-0.188	***	0.017
Muscular/skeletal complaint		-0.073	***	0.003	-0.096	***	0.005
Depression X muscular/ skeletal	127	0.053	***	0.013	0.062	***	0.016
Depression		-0.147	***	0.007	-0.166	***	0.015
Respiratory complaint		-0.032	***	0.004	-0.027	***	0.007
Depression X respiratory complaint	63	0.060	***	0.016	0.032		0.023
Depression		-0.143	***	0.007	-0.164	***	0.015
Urinary complaint		-0.043	***	0.006	-0.040	***	0.009
Depression X Urinary complaint	57	0.039	*	0.006	0.030		0.025
Phobia		-0.097	***	0.009	-0.086	***	0.018
Muscular/skeletal complaint		-0.072	***	0.003	-0.093	***	0.005
Phobia X muscular/skeletal	100	0.038	**	0.016	0.012		0.022
Alcohol dependency		-0.007		0.004	0.0001		0.008
Muscular/skeletal complaint		-0.072	***	0.003	-0.095	***	0.005
Alcohol dependency X muscular/skeletal	176	0.019	*	0.011	0.024		0.015

***p < 0.01, **p < 0.05, *p < 0.1.

### Severity of mental health condition

We have explored the severity of four conditions: depression (n = 367), anxiety (n = 613), phobia (n = 267) and panic (n = 121). We estimate model (1) and in the vector *M*, the dichotomous variable for the presence of anxiety (depression) is replaced with a set of four dummy variables indicating the score, compared to a baseline of zero. The results are shown in Table 6; the effect on other coefficients is negligible so only the estimated coefficients on the new set of dummy variables are reported. The results for depression are as expected, the utility decrement increases with the severity of the condition.

**Table 6:** Impact of severity of Anxiety, Depression, Phobia and Panic on SF-6D and EQ-5D scores

**Depression (n = 367 with depression present)**				
	**Score 1 n = 1047**	**Score 2 n = 75**	**Score 3 n = 219**	**Score 4 n = 73**
SF6 – coefficient	-0.062***	-0.084***	-0.127***	-0.149***
SF6 – standard error	0.004	0.005	0.006	0.010
EQ-5D – coefficient	-0.052***	-0.069***	-0.120***	-0.177***
EQ-5D– standard error	0.007	0.009	0.011	0.021
**Anxiety (n = 613 with anxiety present)**				
	**Score 1 n = 277**		**Score 2 n = 336**	
SF6 – coefficient	-0.047***		-0.070***	
SF6 – standard error	0.003		0.006	
EQ-5D – coefficient	-0.032***		-0.068***	
EQ-5D– standard error	0.006		0.012	
**Phobia (n = 267 with phobia present)**				
	**Score 1 n = 139**		**Score 2 n = 138**	
SF6 – coefficient	-0.017***		-0.039***	
SF6 – standard error	0.004		0.005	
EQ-5D – coefficient	-0.010		-0.012	
EQ-5D– standard error	0.007		0.010	
**Panic (n = 121 with panic present)**				
	**Score 1 n = 57**		**Score 2 n = 64**	
SF6 – coefficient	-0.040***		-0.037	
SF6 – standard error	0.011		0.01	
EQ-5D – coefficient	-0.015		-0.024	
EQ-5D– standard error	0.019		0.016	

***p < 0.01, **p < 0.05, *p < 0.1. "*" refers to p<0.1 or 10% significance level.

For anxiety, while there is a gradient overall from level 1 to 4, the estimated coefficient for levels 3 and 4 have very similar decrements. In this case, the levels have been aggregated to represent only two severity levels with the more severe level showing a higher SF-6D (EQ-5D) decrement of -0.070 (-0.068) compared with the lower severe level of -0.047 (-0.032).

The severity levels for phobia and panic have also been aggregated to distinguish between two severity levels. In the case of phobia, higher decrements are observed in both SF-6D and EQ-5D scores for the more severe levels. It is noted that the EQ-5D scores are not significant. However, for panic disorder both SF-6D and EQ-5D show a lower decrement for the more severe level and only the SF6D value is significant. This is possibly mainly due the fact that there are only 57 and 64 individuals in the less severe and more severe categories respectively.

## Discussion

Our estimates from over 10,000 responses to the APMS 2000 and 2007 show that all but three of the mental health conditions considered here have a statistically significant and relatively large adverse effect on health utility measured by both SF-6D and EQ-5D indices. These effects are larger than for self-reported physical health conditions. Another study also found that HRQoL decrements from mental health conditions are higher than those reported by suffers of chronic physical conditions [1]. Depression, MADD and GAD are the mental health conditions that impact most on health. The three mental health conditions that are not statistically significant are alcohol dependency, personality disorder and psychosis. In considering co-morbidities, we have used interaction terms and in most cases they are significant confirming the fact that we cannot simply add HSUVs decrement of co-morbid health conditions. The impact on HSUVs is generally less than the addition of the separate health conditions. When considering interactions between mental and physical health conditions, the majority are positive, however in one case the interaction is negative and significant suggesting that the presence of both conditions reduces health utility by a larger amount that the additive model would suggest. Brief exploration of the effects of the severity of anxiety and depression on the health utility index, suggest that for depression the results are as expected, the utility decrement increasing with the severity of the condition. However, for anxiety, panic and phobia, only two severity levels were considered.

Utility decrements have been measured using both SF-6D and EQ-5D predicted using a published mapping function. Both measures on the whole depict a similar picture. In general, for most physical and mental health conditions, the decrements shown by SF-6D and EQ-5D are similar and this finding is supported by a study comparing SF-6D and EQ-5D across seven patient groups with physical health conditions [27]. A multi-centre randomised control trial in Netherlands [28] found that EQ-5D utilities led to significant higher health gains even though EQ-5D mean scores were lower than SF-6D scores at baseline. A study on patients with schizophrenia revealed similar EQ-5D and SF-6D mean scores at baseline but higher EQ-5D mean scores higher at follow-up [29].

In model [3], the utility decrements for eye complaints are statistically insignificant. This finding is line with a recent review where it was found that SF-6D and EQ-5D are inadequate measures for such conditions [30]. Similarly, the insignificant coefficient for psychosis is in line with the finding that generic measures are not suitable for use in schizophrenia, a psychotic disorder [31]. An additional reason why personality disorder and psychosis are not statistically significant could be due to relatively small numbers, as less than 1% of the sample has these conditions. For alcohol dependency we explored whether the insignificant result was due to our relatively broad definition of *any* dependency, by testing the effects of moderate and severe dependence on alcohol (as defined by the SADQ) alone, but these were not statistically significant either. Another study found that alcohol dependence was insignificant but similar to this paper, it was not possible to understand how and why this was the case [1].

The effects of co-morbidities are investigated through interaction terms. For the majority of mental health co-morbidities, the interaction terms are positive and often offset the decrement caused by a single condition. While it is understandable that this may be the case, clinically it is difficult to explain. It may be that patients get better at managing their conditions having been exposed to another mental health condition. In the presence of a mental health condition and a physical health condition, some of the results are very surprising. First, several of the interaction terms as measured by EQ-5D score are statistically insignificant even though the both main effects are statistically significant. These include GAD and muscular/skeletal complaints, GAD and digestive complaint, GAD and urinary complaints, MADD and urinary complaints, MADD and skin complaints, depression and respiratory complaints, phobia and muscular/skeletal complaints. This is not the case when the dependent variable is SF-6D. It may be due to the artefact of the mapping approach. In some cases, the main effects are not statistically significant but the interaction terms are. Examples include alcohol dependency and digestive complaints, where alcohol dependency is insignificant but the interaction term is significant. In this case, it is not clear whether it is acceptable to use the HSUVs thus generated. A possible explanation for the latter result is the fact that alcohol may be used to alleviate the symptoms of GAD and MADD.

### Limitations

Overall the models we have estimated have good explanatory power but the RESET test does suggest misspecification problems. We have explored both tobit and generalised linear models for the SF-6D but these were still wrongly specified. The EQ-5D index also suffers from ceiling effects and therefore more sophisticated models may be used to address that. Despite the weaknesses of OLS regressions in the case of SF-6D, it was decided to present the results as they are easy to interpret and in the absence of better estimates, those generated in this paper can be readily used in decision-analytic models.

It is worth stressing here that the data we have used to estimate these models is not based on clinical diagnosis of mental health conditions, but instead on well-established instruments administered by trained interviewers. Furthermore, while data on the mental health conditions are reliable, the data on physical conditions are self-reported and are therefore less reliable since there was no confirmation of the conditions. Finally, the data is limited to those living in private households, though this accounts for the vast majority of people with these conditions.

There may also be a concern as to whether a generic measure of health like the SF-12 adequately assesses the impact of all mental disorders – particularly personality disorders and psychosis. The other limitation is that the EQ-5D estimates are based on a mapping function that been shown to have a tendency to underestimate values at the upper end of the scale and overestimate values at the lower end. Other mapping functions have not overcome this limitation and it may result in an underestimate of the impact of different conditions [32].

## Conclusions

Despite these limitations, this study has a number of interesting findings. First, the estimates provided are based on a representative sample of the UK population. Generally HSUV are generated from trial data and there are always questions as to whether they can be generalised and used outside the trial setting. Therefore the decrements associated with the various mental health conditions and co-morbidities in this paper can be readily used in decision-analytic models in assessing cost-effectiveness of health technologies. Second the APMS is regarded as the most reliable available data in the UK in the area of mental health.

There are several implications of this paper. First, background characteristics need to be controlled for when estimating HSUVs. This is very important but often not accounted in single condition studies. As a result the HSUV decrement associated with a particular health condition is possibly higher which, in turn has implications in cost-effectiveness analysis. Second, the paper has also been able to generate estimates for HSUVs associated with long-term depression. Third, the paper shows that simply adding HSUVs for co-morbid health conditions is not appropriate. Fourth, it provides decrements that can be used to reflect severity of the following conditions: depression, anxiety, panic and phobia. These are useful parameters that can be used in decision analytic models where conditions of patients may be shown to progress.

## Abbreviations

APMS: Adult Psychiatric Morbidity Survey; CIS-R: Clinical Interview Schedule Revised; GAD: Generalised Anxiety Disorder; HSUV: Health State Utility Values; MADD: Mixed Anxiety Depressive Disorder; NICE: National Institute for Health and Care Excellence; OCD: Obsessive Compulsive Disorder; OLS: Ordinary Least Squares; PGLM: Postestimation Generalized Linear Models; QALY: Quality Adjusted Life Year; HRQoL: Health-Related Quality of Life; RESET: Ramsey Regression Equation Specification Error Test; SAD-Q: Severity of Alcohol Dependence Questionnaire; SCAN: Schedules for Clinical Assessment in Neuropsychiatry; SCID – II: Structured Clinical Interview.

## Competing interests

The authors declare that they have no competing interests.

## Authors' contributions

PL, JR and JB designed the study. JR and PL prepared the dataset and carried out initial analysis for the report submitted to the funder. ADK carried out further statistical analyses and wrote the paper. All authors have read and approved the final manuscript.

## Supplementary Material

Additional file 1 **Definition and variables used in the analysis. Figure 1a**: Distribution of SF-6D index. **Figure 1b**: Distribution of EQ-5D index. Click here for file
