# Supplementary material for: Quality of life impact of mental health conditions in England: results from the adult psychiatric morbidity surveys
Source: Health Qual Life Outcomes. 2014 Jan 14;12:6. doi: 10.1186/1477-7525-12-6 (PMC3901021; doi:10.1186/1477-7525-12-6)
Supplement: Additional file 1 — Definition and variables used in the analysis. Figure 1a: Distribution of SF-6D index. Figure 1b: Distribution of EQ-5D index. [file 1477-7525-12-6-S1.docx]

**Additional file 1: Table S1 Variables and Definitions**

| **Variable Name** | **Definition** |
| --- | --- |
| Marital status | A set of dummy variables that take the value of 1 if the respondent is married or widowed/separated/divorced else 0 if the respondent is single. |
| Age | Age in years and age squared. |
| Children | A dummy variable taking the value of 1 if the respondent has a child under age 16. |
| Education | A set of dummy variables that take the value of 1 if the respondent holds any educational qualifications else 0. The categories are: degree, HND/high level vocational qualification, A level, GCSE/O level, lower qualifications. |
| Income | A set of dummy variables that take the value of 1 for various income brackets. These are: £5200 - £10399pa, £10400 -£15559pa, £15600 - £20799pa, £20800 - £33799pa and £33800 or more. The base category is under £5200pa. |
| Ethnicity | A dummy variable takes the value of 1 where the respondent is not white. |
| Employment status | A dummy variable takes the value of 1 if the respondent states he/she is working, else zero. |
| Region | A set of dummy variables that each take the value of 1 if the respondent lives in the region else zero. The regions include, the North, North West, Yorkshire and Humber, East Midlands, West Midlands, East, South West, South East. Greater London is the base category. |
| Year | In models where the data from 2000 and 2007 is pooled, a dummy variable takes the value 1 if the data is from 2007 and 0 for 2000. |
| Physical health | A set of dummy variables that each take the value 1 if the respondent has the health problem, and zero otherwise. The conditions are: muscular/skeletal, respiratory, digestive, heart/circulatory, urinary, skin, ear, eye, neoplasm, blood, infection. The omitted category is no physical health problem. |
| Mental health | 1. A set of dummy variables that each take the value 1 if the respondent has the health problem, and zero otherwise. The conditions are generalised anxiety disorder, mixed anxiety depressive disorder, panic disorder, obsessive compulsive disorder, phobia, psychosis, personality disorder, depression, alcohol dependence and drug dependence. The omitted category is no mental health problem. 2. A set of 7 dummy variables to represent levels of overall CIS-R score. The CIS-R score is an instrument designed to measure neurotic symptoms. The range is from zero to 36+. CISR=2=a score of 6-11; CISR=3 = a score of 12-17; CISR=4 = a score of 18-23; CISR=5 = a score of 24-29; CISR=6 = a score of 30-35; CISR=7 = a score of 36+. The omitted category is CISR=1 which indicates a score of 0-5. |

**Figure 1a: Distribution of SF-6D index**

**Figure 1b: Distribution of EQ-5D index**
